# *In vitro* studies on the cytotoxicity, and elastase and tyrosinase inhibitory activities of marigold (*Tagetes erecta* L.) flower extracts

**DOI:** 10.3892/etm.2013.1373

**Published:** 2013-10-30

**Authors:** OMBOON VALLISUTA, VEENA NUKOOLKARN, AMPOL MITREVEJ, NARONG SARISUTA, PIMPORN LEELAPORNPISID, AMPAI PHRUTIVORAPONGKUL, NUTTANAN SINCHAIPANID

**Affiliations:** 1 Department of Pharmacognosy, Faculty of Pharmacy, Mahidol University, Bangkok 10400, Thailand; 2 Department of Manufacturing Pharmacy, Faculty of Pharmacy, Mahidol University, Bangkok 10400, Thailand; 3 Department of Pharmaceutical Technology, Faculty of Pharmaceutical Sciences, Chiang Mai University, Chiang Mai 50200, Thailand

**Keywords:** *Tagetes erecta* L., elastase inhibitor, tyrosinase inhibitor, H460 cells, Caco-2 cells, marigold flower extracts

## Abstract

Marigold (*Tagetes erecta* L.) has long been used as a medicinal herb for a number of therapeutic activities. In the present study, the cytotoxicities of ethanol and ethyl acetate extracts of marigold flowers and their inhibitory effects on elastase and tyrosinase enzymes were investigated. An MTT assay was performed to measure the cytotoxicity of these two extracts on the H460 lung cancer and the Caco-2 colon cancer cell lines. An elastase assay kit, based on the digestion of a non-fluorescent elastin substrate to highly fluorescent fragments by elastase, was used for the elastase inhibition assay. Tyrosinase inhibition activity was investigated using the dopachrome method with L-3,4-dihydroxyphenylalanine (L-DOPA) as a substrate. The data obtained in this study demonstrated that the extracts were nontoxic to H460 and Caco-2 cell lines. The elastase inhibition activities of ethanol (250 μg/ml) and ethyl acetate (125 μg/ml) extracts were found to be significantly higher than that of the negative control. The tyrosinase inhibition activities of ethanol and ethyl acetate extracts, in terms of the mean inhibition concentration (IC_50_), were 1,078 and 1,467 μg/ml, respectively. To the best of our knowledge, the present study has demonstrated for the first time that marigold flower extracts possess tyrosinase inhibition activity. The activities of ethanol and ethyl acetate extracts of marigold flowers were investigated *in vitro* and indicated that these extracts possess useful properties that may be of interest for cosmetic development.

## Introduction

Marigold (*Tagetes erecta* L.), an ornamental plant, belongs to the *Asteraceae* family and is commonly known as ‘Dow Ruang’ in Thailand. Numerous traditional uses of this plant have been reported. The whole plant has been used to treat bronchitis, rheumatic pain, cold and respiratory diseases, and as a stimulant and muscle relaxer (1). The flowers have been used to treat fevers, epileptic fits, scabies, liver complaints and eye diseases, and have been demonstrated their astringent, carminative and stomachic effects (2). In addition, this plant has been used to cure boils, carbuncles, ulcers, bleeding piles, colds, colic, earaches, headaches, myalgia, stomach aches and rheumatism, and has displayed analgesic and antihyperlipidemic effects (3,4). Although marigold and its extracts have long been used as traditional remedies, the precise biological activities of these remedies have not yet been elucidated.

Elastase is a member of the serine protease enzyme family that hydrolytically degrades elastin, a connective tissue component. The levels of elastase are usually controlled by endogenous inhibitors (5,6). Elastin has been demonstrated to form elastic fibers in the skin dermis, providing elasticity to connective tissues and thus influencing skin elasticity. Damage to the skin results in reduced skin elasticity (7) and the linearity of dermal elastic fibers, thus inducing wrinkling and sagging (8). Therefore, certain elastase inhibitors have been utilized for dermatological preparations to reduce the wrinkling and aging of skin.

Tyrosinase, a copper-containing monooxygenase, is an important enzyme that catalyzes melanin synthesis in melanocytes. The hyperpigmentation of the epidermis and dermis has been demonstrated to depend on either increased numbers of melanocytes or the activity of the tyrosinase enzyme (9). The accumulation of excessive epidermal pigmentation results in various undesirable dermatological disorders, including melasma associated with age, freckling, age spots and sites of actinic damage (10). As the overproduction of melanin is an unexpected phenomenon, tyrosinase inhibitors have become increasingly important in medication and in cosmetics to prevent hyperpigmentation (11,12).

In the present study, ethanol and ethyl acetate extracts of marigold (*Tagetes erecta* L.) flowers were investigated for their cytotoxicity, total phenolic content (TPC) and inhibitory effects on elastase and tyrosinase enzymes.

## Materials and methods

### Chemicals and reagents

The human lung cancer (NCI-H460) and colon cancer (Caco-2) cell lines were obtained from the American Type Culture Collection (Manassas, VA, USA). MTT [3-(4,5-dimethylthiazol-2-yl)-2,5 diphenyltetrazolium bromide] was purchased from Sigma Chemical (St. Louis, MO, USA) and RPMI-1640 medium was obtained from Invitrogen Life Technologies (Carlsbad, CA, USA). The EnzChek^®^ Elastase Assay kit (E-12056) was purchased from Molecular Probes (Eugene, OR, USA), and mushroom tyrosinase, tyrosine and L-3,4-dihydroxyphenylalanine (L-DOPA) were obtained from Sigma-Aldrich Chemie GmbH (Steinheim, Germany). Ethanol (EtOH), ethyl acetate (EtOAc), dimethylsulfoxide (DMSO) and sodium dodecyl sulfate (SDS) were purchased from Merck KGaA (Darmstadt, Germany).

### Plant material

Marigold (*Tagetes erecta* L.) flowers were collected from Chiang Mai, Thailand in the winter of 2008 and authentication was performed by Dr Kongkanda Chayamarit in the Forest Herbarium (Forest Research Office, Royal Forest Department, Bangkok, Thailand). Voucher specimens have been deposited at the Herbarium Section (Northern Research Center for Medicinal Plants, Faculty of Pharmacy, Chiang Mai University, Chiang Mai, Thailand).

### Preparation of marigold extracts

Dried powder of the marigold flower (250 g) was separately extracted by Soxhlet’s apparatus (Sigma-Aldrich, Gillingham, UK) with two different solvents, ethyl acetate and ethanol. Each extract was subsequently filtered through Whatman No.4 paper and evaporated under a vacuum. The residues obtained from the ethyl acetate and ethanol extracts were named MF_EtOAc (yield, 10.26%) and MF_EtOH (yield, 23.83%), respectively. MF_EtOAc and MF_EtOH extracts were employed for further study.

### Determination of TPC

The TPCs of MF_EtOAc and MF_EtOH extracts were determined using the Folin-Ciocalteau method as previously described by Kähkönen *et al*(13). Briefly, 1 ml of the appropriately diluted sample was mixed with 5 ml Folin-Ciocalteau reagent and 4 ml sodium carbonate (7% w/v). The mixture was fully agitated and allowed to stand for 30 min in the dark at room temperature. The absorbance was then measured at 765 nm using a spectrophotometer (Lambda 25; Perkin Elmers, Waltham, MA, USA). The TPC value of each extract was determined in comparison with standard gallic acid and expressed as milligram gallic acid equivalents in 1 g of dried extract (mg GAE/g).

### Cell culture

H460 cells were cultured in RPMI-1640 medium supplemented with 5% fetal bovine serum (FBS), 2 mM L-glutamine, 100 U/ml penicillin and 100 μg/ml streptomycin. The cells were grown in a humidified atmosphere of 5% CO_2_ at 37°C until >60% confluence was reached. The cells were then passaged every other day using a solution containing 0.05% trypsin and 0.5 mM EDTA.

Caco-2 cells were cultured in Dulbecco’s modified Eagle medium to which 10% FBS, 100 U/ml penicillin, 0.1 mg/ml streptomycin and 2 mM L-glutamine were added. The cells were incubated under the same conditions as the H460 cells and subcultured every week using 0.5% trypsin and 0.5 mM EDTA solution.

### Cytotoxicity studies

The cytotoxicity of the MF_EtOH and MF_EtOAc extracts was assessed using the MTT assay. Mitochondrial respiration, an indicator of cell viability, reduced MTT from a yellow water-soluble dye to a purple insoluble formazan product by succinate dehydrogenase (14). Since MF_EtOH and MF_EtOAc extracts are partially water-soluble, DMSO was employed to enhance their solubilities in an aqueous medium. It was found that 0.5% DMSO solution did not significantly affect the cell viability compared with that of the control group (P<0.05). Therefore, test solutions of MF_EtOH and MF_EtOAc extracts were prepared in 0.5% DMSO aqueous solution. The toxicity of the MF_EtOH extract was studied at three concentrations (10, 50 and 100 μg/ml), while the toxicity of the MF_EtOAc extract was studied at two concentrations (10 and 50 μg/ml) due to the lower solubility.

Caco-2 and H460 cells were cultured separately in 96-well plates for 48 and 24 h at seeding densities of 5×10^4^ and 2×10^4^ cells/well in 100 μl medium, respectively. Prior to testing, the cell culture medium was removed and cells were washed with phosphate-buffered saline (PBS). Experiments were initiated by adding 100 μl MF_EtOH or MF_EtOAc solution to each well. After 24 h of incubation, cells were washed with PBS and 100 μl MTT solution (0.5 mg/ml) was added to each well. Following a further 4 h of incubation, 100 μl SDS solution (10% w/v) was added to each well and the cells were incubated overnight. Absorbance was then measured at a wavelength of 590 nm using a Thermomax microplate reader (Molecular Devices, Sunnyvale, CA, USA). Untreated cells (0.5% DMSO) were used as the control group.

### Elastase inhibition activity

The elastase inhibition activities of MF_EtOH and MF_EtOAc extracts were assayed using the EnzChek^®^ Elastase Assay kit. This kit consisted of DQ™ elastin from bovine neck ligaments that was labeled with fluorescent BODIPY^®^ FL conjugate, elastase from pig pancreas, N-methoxysuccinyl-ala-ala-pro-val-chloromethyl ketone (elastase inhibitor) and reaction buffer.

The principle was based on the digestion of non-fluorescent elastin substrate (BODIPY^®^ FL conjugated with DQ™ elastin) to highly fluorescent fragments by elastase. The digestion products were subsequently measured using a fluorescence microplate reader (Thermomax; Molecular Devices).

DMSO solution (5% v/v) was employed to enhance the solubility of MF_EtOH and MF_EtOAc extracts, respectively. The elastase inhibition activity of MF_EtOH was studied at three concentrations (50, 125 and 250 μg/ml), while two concentrations of MF_EtOAc extract (50 and 125 μg/ml) were studied due to the limited solubility of MF_EtOAc. The test was performed in 96-well plates that were hidden from the light. DMSO (5%) and elastase inhibitor were used as a negative and a positive control, respectively.

Following 2 h of incubation at 25°C, fluorescence absorbance was measured at an excitation wavelength of 485 nm and an emission wavelength of 535 nm, in accordance with the instructions supplied with the EnzChek^®^ Elastase Assay kit and described by Leu *et al*(15). The percentage of elastase inhibition activity was calculated using the following equation (1):

$$\text{Elastase inhibition }(\%)=\left(1-\frac{B}{A}\right)\times 100$$

Where ‘A’ was enzyme activity in absence of the inhibitor and ‘B’ was enzyme activity in the presence of the inhibitor.

### Tyrosinase inhibition activity

The tyrosinase inhibition activities of MF_EtOH and MF_EtOAc extracts were determined using the commercially available mushroom tyrosinase as previously described (16). L-DOPA and kojic acid were utilized as the substrate and positive control, respectively. Briefly, 40 μl L-DOPA (0.85 μM) solution was mixed with 40 μl extract solution or kojic acid. A total of 40 μl tyrosinase solution (520 U/ml) was then added to the mixture and the enzyme reaction was evaluated by the absorbance measurement of dopachrome formation at 492 nm using a Thermomax microplate reader (Molecular Devices).

The percentage of tyrosinase inhibition activity was calculated using the following equation (2):

$$\text{Tyrosinase inhibition }(\%)=\left(1-\frac{B}{A}\right)\times 100$$

Where ‘A’ was enzyme activity in absence of the inhibitor and ‘B’ was enzyme activity in the presence of inhibitor. The concentration giving 50% tyrosinase inhibition activity (IC_50_) was determined by interpolation of the dose-response curves.

### Statistical analysis

Results are presented as the mean ± standard deviation. An unpaired Student’s t-test (two-tailed) was used to test the significance of the difference between two mean values. One-way analysis of variance was used when more than two means were compared. P<0.05 was considered to indicate a statistically significant difference.

## Results

### Determination of TPCs

**Figure 1:**
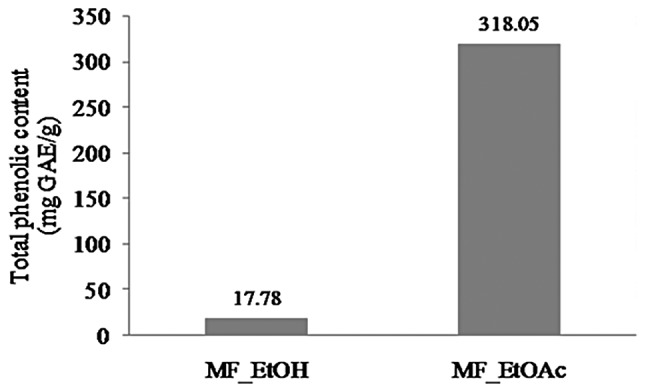
Total phenolic contents of MF_EtOH and MF_EtOAc extracts. MF_EtOH, ethanol extract of marigold flowers; MF_EtOAc, ethyl acetate extract of marigold flowers; GAE, gallic acid equivalents.

The TPCs of MF_EtOH and MF_EtOAc extracts, expressed as gallic acid equivalents, are shown in Fig. 1. Different extraction solvents differed in polarities and therefore resulted in different TPC values of the two extracts. As a result, there was a significant difference between the TPC values of the MF_EtOAc extract (318.05 mg GAE/g) compared with that of the MF_EtOH extract (17.78 mg GAE/g). The TPC value of the MF_EtOAc extract was found to be ~18-fold higher than that of the MF_EtOH extract.

### Cytotoxicity studies

To investigate whether the MF_EtOH and MF_EtOAc extracts affected the viability of H460 and Caco-2 cells, the MTT assay was performed. The cytotoxicity of the MF_EtOH and MF_EtOAc extracts is presented as a percentage of cell viability relative to the control group (100%). The mean and standard deviation of each experiment were calculated from 10 replicates.

**Figure 2:**
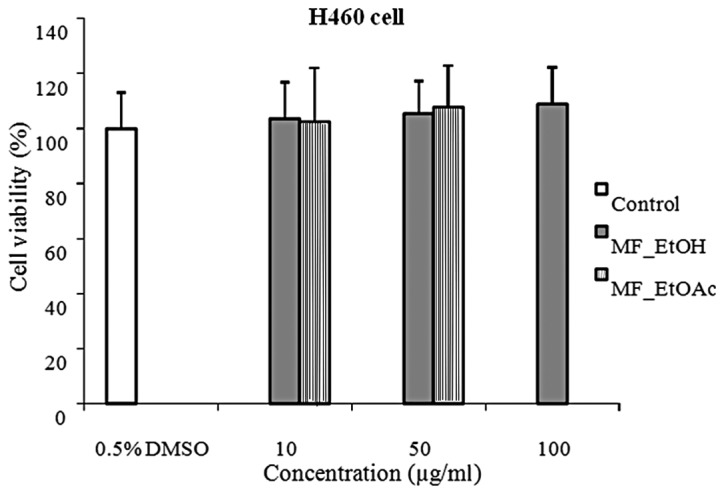
Viability of H460 cells following treatment with MF_EtOH and MF_EtOAc extracts compared with the control group (n=10). MF_EtOH, ethanol extract of marigold flowers; MF_EtOAc, ethyl acetate extract of marigold flowers.

The viability of the H460 cells following treatment with different concentrations of MF_EtOH or MF_EtOAc extracts ranged between 87.78 and 126.2% (Fig. 2). No significant differences were identified among the H460 cell viabilities of the treated and control groups (P>0.05).

**Figure 3:**
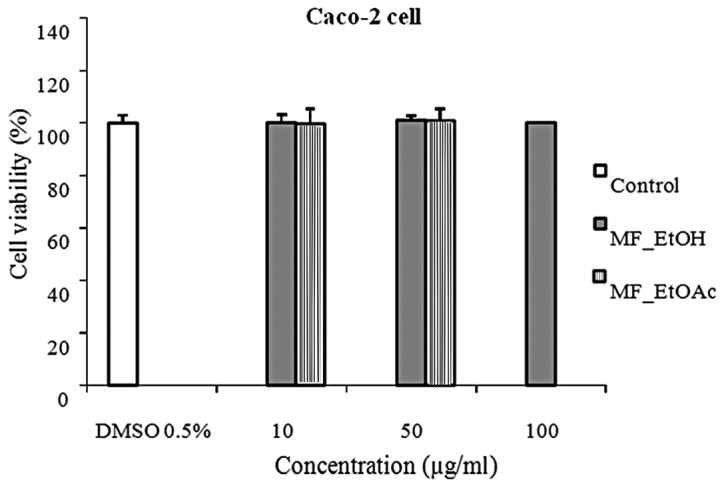
Viability of Caco-2 cells following treatment with MF_EtOH and MF_EtOAc extracts compared with that of the control group (n=10). MF_EtOH, ethanol extract of marigold flowers; MF_EtOAc, ethyl acetate extract of marigold flowers.

The cytotoxicity studies of MF_EtOH and MF_EtOAc extracts on Caco-2 cells obtained similar results to the H460 cells (Fig. 3). The Caco-2 cell viabilities following treatment with different concentrations of MF_EtOH and MF_EtOAc extracts were found to range between 91.3 and 101.9%, with no significant differences among the data groups (P>0.05).

### Elastase inhibition activity

**Figure 4:**
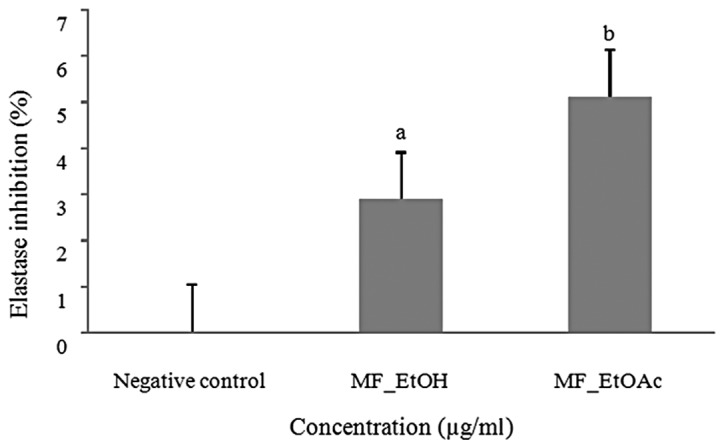
Elastase inhibition activity of MF_EtOH (250 μg/ml) and MF_EtOAc (125 μg/ml) extracts compared with that of the negative control (n=3). ^a^P<0.05, ^b^P<0.01 compared with negative control. MF_EtOH, ethanol extract of marigold flowers; MF_EtOAc, ethyl acetate extract of marigold flowers.

As shown in Fig. 4, the elastase inhibition activities of MF_EtOH and MF_EtOAc extracts were found to be significantly higher than that of the negative control at concentrations of 250 and 125 μg/ml, respectively. When these two extracts were applied at lower concentrations, no significant differences were identified in elastase inhibition activities compared with that of the negative control (data not shown). At a concentration of 125 μg/ml, the elastase inhibition activity of MF_EtOAc extract was observed to be significantly higher than that of 250 μg/ml MF_EtOH extract (P=0.004). It may be inferred that the MF_EtOAc extract possessed stronger elastase inhibition activity than that of the MF_EtOH extract. The positive control showed 82.20% inhibition of elastase activity.

### Tyrosinase inhibition activity

**Figure 5:**
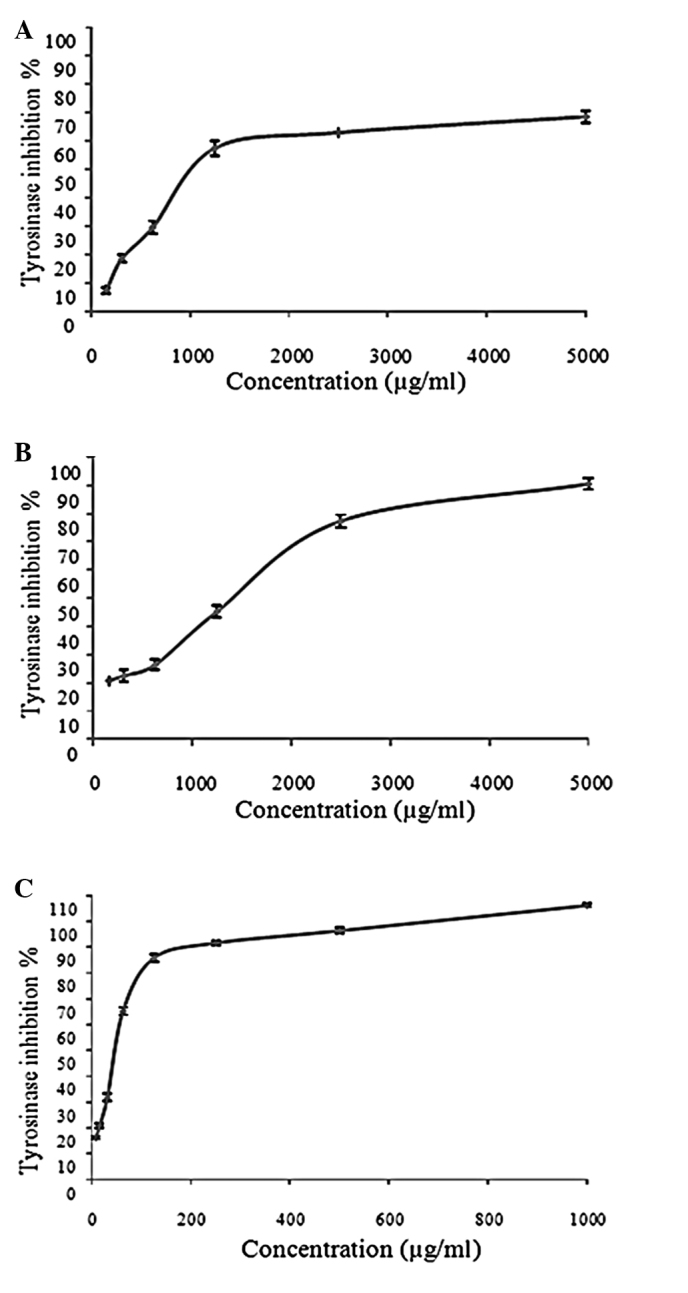
Tyrosinase inhibitory activity of (A) MF_EtOH and (B) MF_EtOAc extracts and (C) kojic acid (n=3). MF_EtOH, ethanol extract of marigold flowers; MF_EtOAc, ethyl acetate extract of marigold flowers.

The *in vitro* inhibition effects of various concentrations of MF_EtOH and MF_EtOAc extracts on tyrosinase activities are shown in Fig. 5. The results demonstrated that MF_EtOH and MF_EtOAc extracts exhibited potent inhibitory effects against mushroom tyrosinase. The IC_50_ values of MF_EtOH and MF_EtOAc extracts against tyrosinase were 1,078 and 1,467 μg/ml, respectively. These observations suggested that the MF_EtOH extract exerted a more marked tyrosinase inhibition compared with that of the MF_EtOAc extract. However, at a concentration of 5,000 μg/ml, MF_EtOAc extract resulted in a higher tyrosinase inhibition of 90.6%, while the MF_EtOH extract exhibited 68.5% inhibition.

Due to the aforementioned tyrosinase inhibition activity, kojic acid was selected as the positive control in this study. The IC_50_ values of MF_EtOH and MF_EtOAc extracts were 23- and 31-fold higher than that of kojic acid (IC_50_=47 μg/ml).

## Discussion

The phenolic compounds present in marigold flower extracts have previously been reported to markedly contribute to the antioxidant capacities of the extracts (17). As an additional study has provided further evidence for this correlation (18), the antioxidant activities of MF_EtOAc and MF_EtOH extracts were not investigated in the present study. According to this correlation, it may be inferred that MF_EtOAc extract, which has a higher TPC, is likely to exhibit a stronger antioxidant activity than that of the MF_EtOH extract.

According to the ISO 10993-5 International Standard (19), a reduction in cell viability by >30% is considered a cytotoxic effect. Following this criterion, MF_EtOH extract at concentrations of 10, 50 and 100 μg/ml and MF_EtOAc extract at concentrations of 10 and 50 μg/ml may be considered to be nontoxic to human lung cancer (H460) and colon cancer (Caco-2) cells.

Elastase, a member of the proteinase family, is mainly responsible for degrading elastin, a connective tissue component. Elastase activity increases with age, resulting in a reduction in elastin and further decreasing the elasticity of skin, which causes the appearance of wrinkles and stretchmarks (8). Therefore, inhibition of elastase activity may be a valuable method to protect against skin aging. For this reason, the marigold flower extracts MF_EtOH and MF_EtOAc, which were found to possess elastase inhibition activities, may be used to prevent the loss of skin elasticity and thus reduce skin wrinkling, sagging and aging.

Although the tyrosinase inhibitory activities of MF_EtOH and MF_EtOAc extracts were significantly lower than that of kojic acid (P<0.05), the present study was, to the best of our knowledge, the first to demonstrate that ethanol and ethyl acetate extracts from marigold flowers possess tyrosinase inhibition activities.

At present, tyrosinase inhibitors have important applications in cosmetics. For clinical usage, tyrosinase inhibitors are employed to treat dermatological disorders associated with melanin hyperaccumulation, and are essential in cosmetics for depigmentation (20). Therefore, MF_EtOH and MF_EtOAc extracts with tyrosinase inhibition activities may be further investigated for their application in skin lightening.

Since no cells were utilized in the elastase and tyrosinase inhibition assays, the solubility of MF_EtOH and MF_EtOAc extracts was able to be significantly enhanced using higher concentrations of DMSO. Therefore, while the cytotoxicity of these two extracts was studied at the maximum concentration of 100 μg/ml, the concentrations of the two extracts were as high as 250 and 5,000 μg/ml in the elastase and tyrosinase inhibition studies, respectively.

The present study demonstrated that MF_EtOH and MF_EtOAc extracts possess elastase and tyrosinase inhibition effects and antioxidant activities. At present, numerous cosmetic companies utilize elastase, melanogenesis inhibitors and antioxidant ingredients in antiwrinkle, skin-lightening and antiaging products. Therefore, MF_EtOH and MF_EtOAc extracts have potential for use in cosmetic products in the future. Moreover, MF_EtOH and MF_EtOAc are plant extracts and, as a result, their use in cosmetic products may be particularly advantageous due to the relatively low incidence of side effects.

In conclusion, the results obtained in the present study demonstrated that ethanol and ethyl acetate extracts from marigold (*Tagetes erecta* L.) flowers were nontoxic to two human cell lines, lung cancer (H460) and colon cancer (Caco-2). Furthermore, the potential of these two extracts as sources of elastase and tyrosinase inhibitors was suggested. The *in vitro* activities of ethanol and ethyl acetate extracts from marigold flowers indicated that these two extracts possess useful properties and may be suitable for cosmetic applications as antiwrinkle, skin-lightening and antioxidant additives. Further studies may focus on evaluating the biological activities of marigold extracts *in vivo* for cosmetic development.
